# Marginal Bone Loss and Peri-Implant Clinical Outcomes of Internal Hexagonal and Internal Conical Implant Abutment Connections: A Narrative Review

**DOI:** 10.7759/cureus.116544

**Published:** 2026-09-19

**Authors:** Sameera Y, Mohan Valiathan, Peter Devadoss, Kamala Kannan Raja, Pretham K Christopher

**Affiliations:** 1 Prosthodontics and Crown and Bridge, Bharath Institute of Higher Education and Research, Chennai, IND; 2 Periodontics, Bharath Institute of Higher Education and Research, Chennai, IND; 3 Prosthodontics and Crown and Bridge, Air Force Institute of Dental Sciences, Bangalore, IND; 4 Prosthodontics and Crown and Bridge, Karpaga Vinayaga Institute of Dental Sciences, Chengalpattu, IND; 5 Dentistry, Chris Dental Center, Chenniai, IND

**Keywords:** dental implants, implant–abutment connection, internal conical connection, internal hexagonal connection, marginal bone loss, morse taper connection, peri-implant outcomes

## Abstract

The implant-abutment connection is the mechanical and biological interface between the implant and the prosthesis, and its geometry influences mechanical stability, sealing of the microgap, stress distribution, and the peri-implant tissue response. This narrative review compares internal hexagonal connections with internal conical connections, of which the Morse taper is a specific deep-cone, friction-locking type, focusing on marginal bone loss and peri-implant clinical outcomes, and distinguishes laboratory and mechanistic evidence from clinical evidence. Laboratory, finite element, and microbiological studies suggest that conical connections may reduce micromovement, microgap opening, and bacterial and endotoxin leakage and may transmit occlusal load more favorably than screw-retained internal hexagonal connections. Systematic reviews and meta-analyses report that internal connections, and conical connections in particular, are associated with lower short- to medium-term marginal bone loss, yet several randomized controlled trials, including a 2025 trial, show no significant difference between connection types in marginal bone loss, peri-implant tissue health, complications, or survival, and both designs achieve high survival. Clinical outcomes are strongly influenced by implant placement depth, abutment height, bone quality, soft-tissue phenotype, platform switching, loading protocol, prosthesis design, oral hygiene, smoking, systemic health, and follow-up duration. On current evidence, connection-specific clinical superiority has not been consistently established. Internal conical connections may be considered when marginal bone preservation, interface stability, and esthetic stability are priorities, whereas internal hexagonal connections may be preferred when prosthetic flexibility, indexed positioning, and retrievability are required. Connection selection should be individualized and guided by patient risk, site factors, prosthetic requirements, and the evidence for the specific implant system rather than by connection geometry alone.

## Introduction and background

Osseointegrated dental implants are a predictable and widely used treatment for partial and complete edentulism, with long-term studies reporting high survival, but crestal bone remodeling and mechanical performance remain decisive for lasting success [1]. Contemporary practice has largely moved from external toward internal implant-abutment connections that aim to improve abutment seating, anti-rotational stability, load transfer, and maintenance of the peri-implant tissues. Two internal designs dominate: the internal hexagonal connection, which uses a hexagonal index for anti-rotational stability and is retained mainly by an abutment screw, and the internal conical connection, in which a tapered abutment engages the internal walls of the implant. The Morse taper connection is a specific internal conical design characterized by a deep, self-locking cone that produces frictional retention; the terms are related but not interchangeable, and this review uses “internal conical” as the general category and “Morse taper” for the deep-cone subtype [2-4].

The configuration of the implant-abutment interface is clinically relevant because it can influence micromovement, bacterial penetration, inflammatory activity, stress distribution, and marginal bone behavior, all of which bear on implant stability, soft-tissue health, esthetics, and the risk of peri-implant disease [2,3]. After placement and loading, physiologic crestal remodeling occurs as the peri-implant tissues establish a supracrestal attachment comprising sulcular epithelium, junctional epithelium, and a connective-tissue zone [5]. Because two-piece systems always incorporate a microgap at the interface, the position and sealing of that interface relative to the crestal bone may modulate the local inflammatory and mechanical environment; in an in vitro comparison, bacterial leakage occurred in 5 of 10 internal hexagonal assemblies versus 2 of 10 Morse taper assemblies, although the difference was not statistically significant [6].

Whether the mechanistic and short-term differences attributed to connection geometry translate into clinically meaningful long-term benefits remains debated. This review examines the structural differences between internal hexagonal and internal conical connections (Figure 1), synthesizes the laboratory and clinical evidence separately, and considers marginal bone loss, peri-implant tissue health, mechanical and prosthetic complications, and implant survival, together with the patient-, implant-, and prosthesis-related factors that modify these outcomes and their implications for connection selection. 

**Figure 1:**
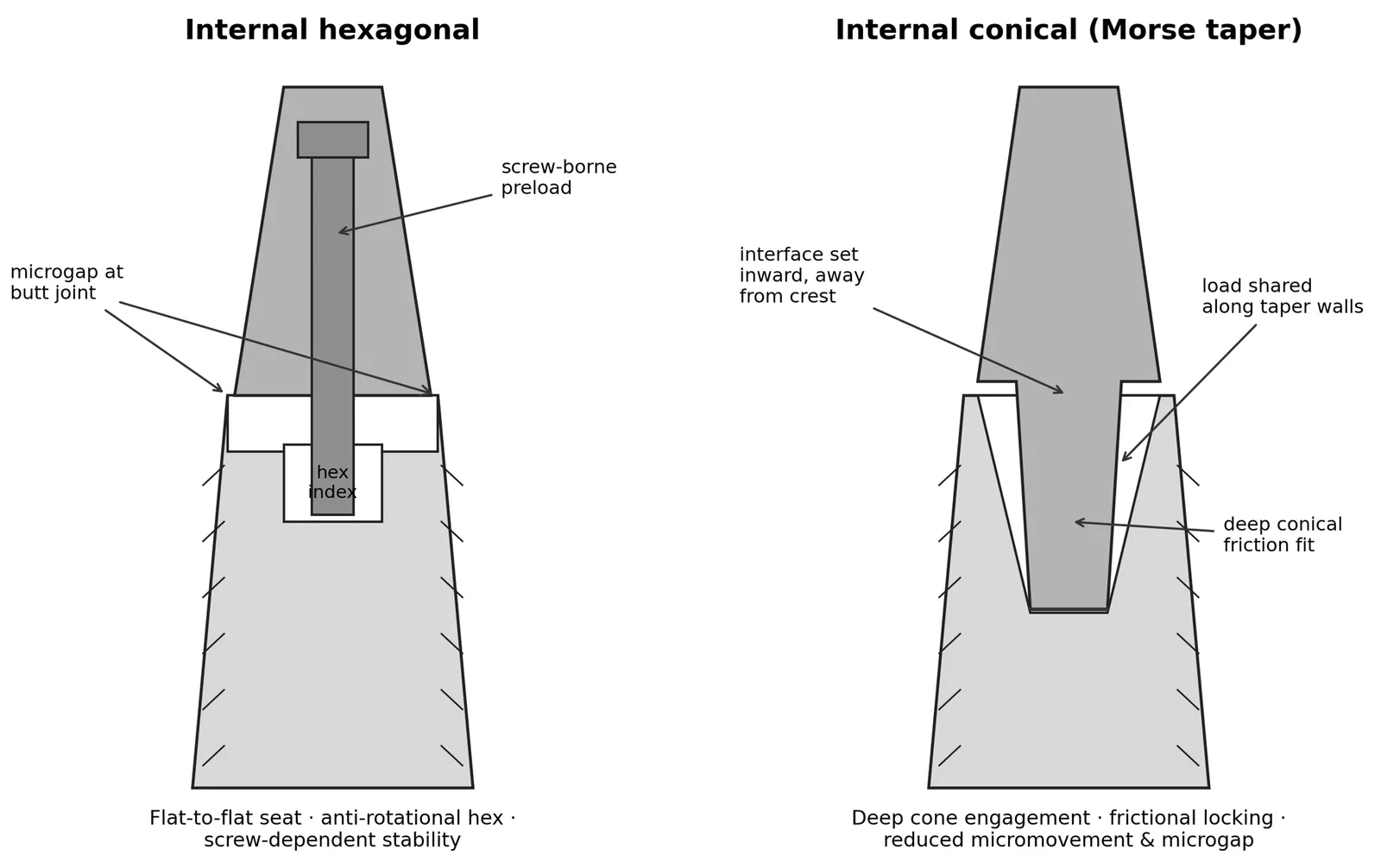
Structural differences between internal hexagonal and internal conical (Morse taper) implant–abutment connections The schematic illustrates differences in connection geometry, interface configuration, stabilization mechanism, and load transfer between the two designs. Credit: Authors’ own illustration created using Microsoft PowerPoint (Microsoft Corporation, Redmond, Washington).

## Review

Literature search

This narrative review synthesizes evidence identified by searching PubMed/MEDLINE, Scopus, Web of Science, Embase, and Google Scholar for English-language articles published between January 2000 and July 2026, using combinations of the terms dental implant, implant-abutment connection, internal hexagon, internal conical, Morse taper, conical connection, platform switching, marginal bone loss, and peri-implant outcomes; reference lists of relevant articles were also screened. Primary clinical studies (randomized and non-randomized trials and prospective and retrospective cohorts) provided the principal clinical evidence, supported by microbiological, biomechanical, and finite element studies for mechanistic context and by systematic reviews and meta-analyses for synthesis and background [3,7,8]. As a narrative review, it did not follow a formal protocol, apply a risk-of-bias appraisal, or perform a quantitative meta-analysis; the aim was a broad, balanced overview rather than a pooled estimate. The identified literature was screened for relevance to implant-abutment connection design, marginal bone loss, peri-implant clinical outcomes, and mechanical or biological complications. Priority was given to systematic reviews, meta-analyses, randomized controlled trials, comparative clinical studies, and relevant biomechanical and microbiological investigations. Reference lists of key articles were also reviewed to identify additional relevant publications. As this was a narrative review, study selection was based on relevance and clinical or scientific contribution rather than a formal systematic screening or risk-of-bias assessment. Publications were excluded when they were unrelated to implant-abutment connection design or did not provide clinically or scientifically relevant information for the review objectives.

Implant-Abutment Connection Designs: Classification and Terminology

Implant-abutment connections are broadly classified as external or internal. External connections, typically an external hexagon, locate the anti-rotational feature above the implant platform and rely on the abutment screw for retention and load resistance; they offer versatility and easy component handling but concentrate functional load on the screw and are comparatively prone to screw loosening and microgap micromovement. Internal connections place the engaging features within the implant body and are further divided into internal flat-to-flat (butt-joint) designs, of which the internal hexagon is the most common, and internal conical designs, in which a tapered abutment seats against the internal walls. The Morse taper is a deep conical connection whose frictional (cold-weld) engagement produces a self-locking fit. Conical connections may be non-indexed, allowing free rotational positioning, or indexed, adding an anti-rotational feature for single-unit restorations and prosthetic orientation [4,9,10]. A related design concept, platform switching, uses an abutment narrower than the implant platform to move the interface inward and has been associated with reduced marginal bone loss [11]. Meta-analytic data suggest that internal connections, and conical connections in particular, tend to preserve crestal bone better than external connections in the short to medium term [3,7], although the magnitude and clinical relevance of these differences remain uncertain. The principal designs and their reported tendencies, each discussed and referenced in the sections that follow, are summarized in Table 1.

**Table 1:** Principal implant–abutment connection designs and their reported features The connection types, key features, and reported tendencies summarized in this table are derived from the literature on implant–abutment connection design and performance [4-11].

Connection type	Key features	Reported tendencies
External hexagon	Anti-rotation above the platform; screw-retained	Versatile and easy to handle; higher screw loosening; more microgap micromovement
Internal hexagon (flat-to-flat)	Internal index; screw-retained; anti-rotational	Predictable; stability depends on screw preload; retrievable
Internal conical (non-Morse)	Tapered-wall engagement	Improved seating; reduced micromovement relative to butt-joint
Morse taper (deep cone)	Deep self-locking cone; frictional retention	Low micromovement; better sealing; possible short-to-medium-term bone preservation
Indexed conical	Cone plus anti-rotational index	Anti-rotation for single units; smaller conical contact area
Platform switching (modifier)	Abutment narrower than the implant platform	Associated with reduced marginal bone loss

Biomechanical Considerations

The mechanical behavior of an implant-abutment assembly reflects how occlusal forces are transmitted across the interface, and much of what is known derives from laboratory and finite element studies that describe potential rather than demonstrated clinical effects [4,9,10]. In the internal hexagonal connection, a hexagonal index and retaining screw hold the abutment, and joint stability depends heavily on screw preload; axial forces are borne partly by the platform, but oblique forces generate bending moments that load the screw and concentrate stress at the crestal cortical bone [9]. In the internal conical connection, the tapered walls engage over a larger area and transfer load through frictional contact, reducing reliance on the screw; friction-fit conical connections show very limited rotation and horizontal movement under load in vitro, although stability varies with implant system, taper angle, and manufacturing tolerances [4]. Finite element analyses indicate that a Morse taper geometry can produce less abutment-screw separation than comparison designs [12].

When functional forces exceed the stability provided by screw preload and interfacial friction, micromovement occurs and can widen the microgap, increase component wear, reduce preload, and permit microbial penetration [13-15]. Screw loosening arises when the separating forces during function exceed the residual clamping force; surface microroughness flattens under an initial settling effect, and repeated axial, oblique, and rotational loading can further reduce preload, particularly with inadequate torque or unbalanced occlusion [13,14]. Fatigue testing shows that repeated loading can deform the screw or cause joint failure, with resistance differing among interface designs [13], and cyclic loading of implant-abutment assemblies reduces removal torque in a design- and fit-dependent manner [14]. Comparative cyclic testing has shown that abutment displacement and connection stability differ under axial, torsional, and off-axis loading [10], and, in direct comparisons, conical connections generally display greater stability and lower microleakage than internal hexagonal connections under cyclic fatigue [15].

Platform switching may redistribute mechanical and inflammatory stimuli away from the crestal bone and is independently associated with better marginal bone maintenance; therefore, its presence should be considered when attributing bone-level differences to connection geometry [11]. Collectively, these data indicate a plausible biomechanical advantage for conical connections in resisting micromovement and screw loosening, but they characterize mechanisms and bench performance rather than clinical endpoints.

Biological and Microbiological Considerations

Every two-piece implant incorporates a microgap at the abutment interface that can harbor microorganisms and their products [16-18]. In vitro, conical assemblies permit less bacterial penetration than screw-retained flat-to-flat connections [16], consistent with the lower leakage observed for Morse taper joints [6]; however, conical joints are not hermetic, and molecular studies have demonstrated endotoxin transport across internal conical interfaces [17]. Functional micromovement can create a pumping effect that moves bacteria and inflammatory products between the interface and the surrounding tissues [8,16-18].

Microbial contamination and mechanical irritation activate an inflammatory infiltrate of neutrophils, macrophages, and lymphocytes, with pro-inflammatory mediators that can contribute to connective-tissue breakdown and osteoclastic bone resorption [8,18]. Experimentally, an inflammatory cell accumulation forms around the implant-abutment junction, and a subcrestally positioned interface produces a higher inflammatory cell density than a supracrestal one [18], suggesting that the distance between the contaminated interface and the crest is as important as the microgap itself. The connection and abutment may thus act as predisposing or precipitating factors for peri-implant disease, alongside plaque accumulation and host susceptibility [8].

The peri-implant mucosa attempts to seal the bone by forming a supracrestal attachment whose quality depends on abutment surface, handling, and material [19]; a stable, well-adapted seal limits apical microbial migration, whereas repeated abutment disconnection or micromotion impairs adhesion and increases inflammation. Crestal bone change appears more closely related to micromotion than to microgap size alone [20]. Collectively, these mechanistic pathways (Figure 2) indicate why connection geometry could influence marginal bone, but they do not by themselves establish a clinical difference.

**Figure 2:**
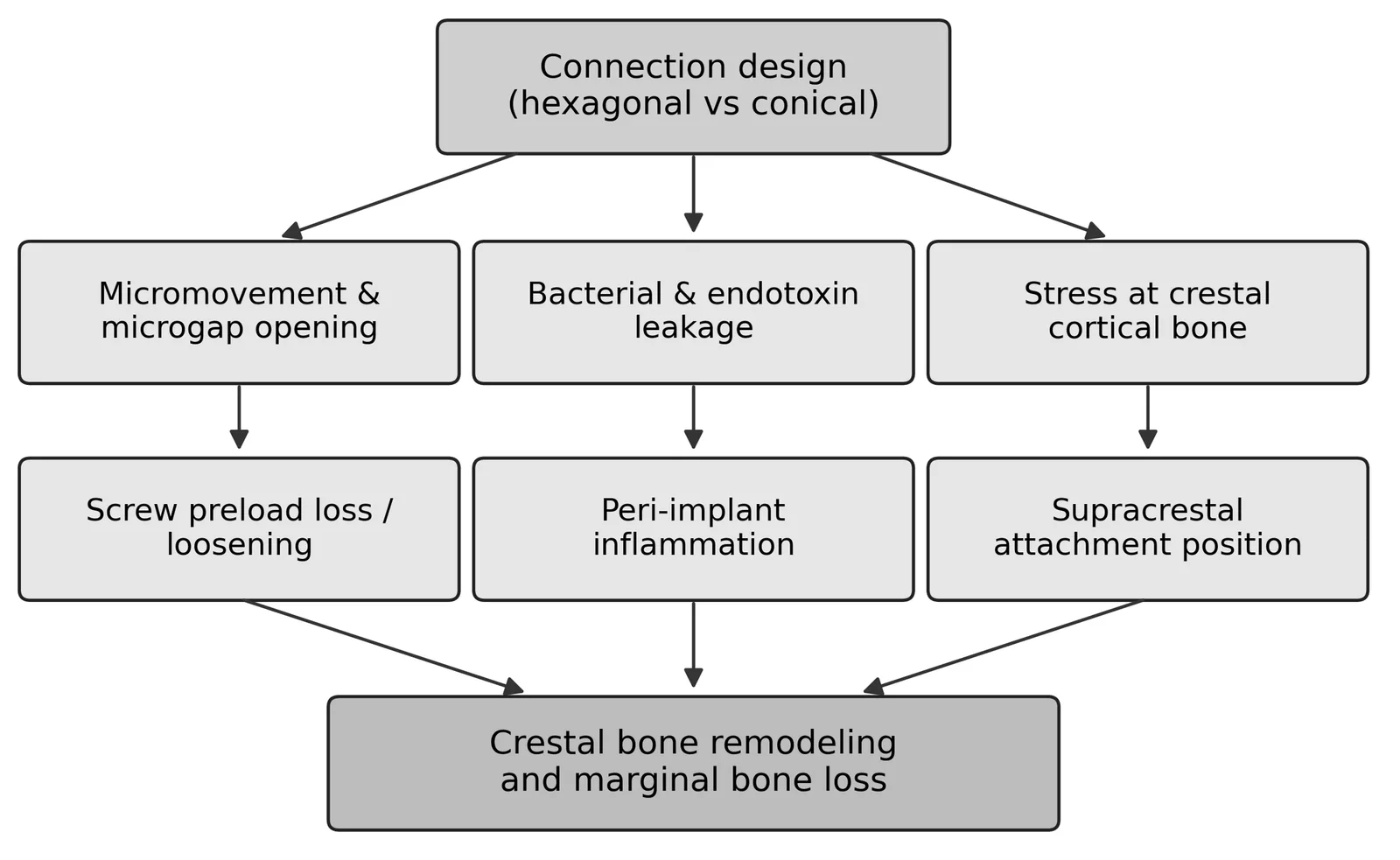
Biomechanical and biologic pathways linking implant–abutment design with marginal bone loss These pathways derive mainly from laboratory and finite element studies and represent potential mechanisms rather than demonstrated clinical effects. Credit: Authors’ own illustration created using Microsoft PowerPoint (Microsoft Corporation, Redmond, Washington).

Marginal Bone Loss: Definitions, Measurement, and Evidence

Marginal (crestal) bone loss is measured radiographically at the mesial and distal aspects of the implant relative to a reproducible baseline, usually implant placement, prosthesis delivery, or loading. Some early loss is physiologic remodeling, whereas progressive loss accompanied by bleeding, suppuration, or increasing probing depth signals peri-implant disease [21]. A historically cited success criterion proposed no more than about 1.5 mm of loss in the first year and less than 0.2 mm annually thereafter [22]; this threshold predates contemporary implant surfaces, platform switching, and conical interfaces and is best regarded as a historical reference rather than a universal contemporary standard. Marginal bone level is typically monitored on standardized intraoral radiographs obtained at defined intervals.

Synthesized evidence suggests a connection effect on marginal bone. A systematic review and meta-analysis concluded that crestal bone levels are better maintained in the short to medium term with internal interfaces, with conical connections appearing most advantageous, while cautioning that further study is needed [3]. A separate meta-analysis comparing external and internal connections likewise favored internal designs for bone preservation, although the authors emphasized heterogeneity among the included studies [7].

Primary comparative studies are more mixed. In a 12-month randomized pilot trial, mean marginal bone loss was lower for the internal conical connection (0.25 mm) than for the internal hexagonal connection (0.70 mm) [23]. A retrospective study of 540 implants reported significantly lower loss for the internal cone connection (0.68 versus 0.99 mm at 12 months; 0.78 versus 1.12 mm at 24 months), but the two groups used different implant systems, so the connection effect cannot be isolated [24]. An earlier retrospective analysis similarly linked connection configuration to crestal bone level [2]. In contrast, a multicenter randomized trial of 90 patients found no significant difference at one year [25], and its five-year report found comparable loss (1.41 mm conical versus 1.38 mm hexagonal; p = 0.75) [26]; a three-year study also found no significant difference between internal hexagonal and internal conical implants [27], and a 2025 randomized trial reported no significant effect of connection design on marginal bone loss [28]. Overall, meta-analyses and some short-term or retrospective studies favor conical connections, whereas the randomized trials that match other variables generally show no clinically significant difference. Key comparative studies are summarized in Table 2.

**Table 2:** Summary of key comparative studies of implant–abutment connection type and marginal bone loss MBL, marginal bone loss; RCT, randomized controlled trial.

Study (year)	Design	Connections	Follow-up	Key finding
Caricasulo (2018) [3]	Systematic review/meta-analysis	Connection types	—	Conical favored (short-medium term)
Lemos (2018) [7]	Systematic review/meta-analysis	External vs internal	—	Internal favored for bone preservation
Galindo-Moreno (2021) [23]	RCT (pilot)	Internal hex vs internal conical	12 months	Lower MBL with conical (0.25 vs 0.70 mm)
Szyszkowski (2019) [24]	Retrospective	Internal cone vs internal hex (different systems)	Up to 5 years	Lower MBL with cone; systems differed
Cannata (2017) [25]	Multicenter RCT	Conical vs internal hex	1 year	No significant difference
Grandi (2021) [26]	Multicenter RCT	Conical vs internal hex	5 years	Comparable (1.41 vs 1.38 mm; p=0.75)
Mihali (2021) [27]	Clinical (3-year)	Internal hex vs conical	3 years	No significant difference
Rubianes-Porta (2025) [28]	RCT	External/internal hex vs conical	Posterior single-tooth	No significant effect on MBL
Geckili (2017) [29]	Clinical	Screwless Morse taper vs screw-retained	1 year	No significant difference

Peri-Implant Clinical Outcomes

Beyond radiographic bone level, peri-implant health is assessed with the plaque index, bleeding on probing, probing depth (interpreted together with restoration contour, tissue thickness, and probing force), mucosal recession, and keratinized mucosa width. Peri-implant health is defined by the absence of inflammation; peri-implant mucositis by bleeding and inflammation without progressive bone loss; and peri-implantitis by inflammation combined with progressive bone loss beyond initial remodeling [21]. Progressive increases in probing depth with bleeding and suppuration are more informative than any single measurement.

A systematic review and meta-analysis found that although reduced keratinized mucosa width is associated with poorer peri-implant health indicators, its role as an independent risk factor for peri-implant disease is inconclusive, particularly with adequate maintenance [30]. Connection design may also influence peri-implant soft-tissue dimensions; a 12-month randomized study reported greater soft-tissue gain and less recession around conical than butt-joint connections [31]. Although the connection and abutment may act as predisposing or precipitating factors for peri-implant disease [8], neither design prevents plaque-associated disease; a conical interface may offer better sealing and less micromotion in theory, but clinical peri-implant outcomes are governed mainly by hygiene, prosthetic contour, mucosal thickness, smoking, and maintenance.

A prospective study of Morse taper implants reported high stability, favorable tissue conditions, and predictable survival [32]. In randomized comparisons, both connections showed comparable peri-implant conditions, complication rates, and survival at one year [25] and at five years [26], and survival was high in the cited studies [25,26,32].

Mechanical and Prosthetic Complications

Mechanical and prosthetic complications can compromise an implant-supported restoration even after successful osseointegration. Screw loosening is the most common technical complication of screw-retained connections and reflects loss of preload under function; because internal hexagonal connections rely on screw preload, interfacial micromovement can accelerate torque loss and loosening, whereas conical connections share load through frictional contact and are generally less susceptible [14,15]. Screw or abutment fracture is less frequent but more serious and is associated with repeated loosening, reduced component dimensions, unfavorable crown-to-implant ratios, cyclic fatigue, and bruxism [13,14,33]. Conical connections are not complication-free: in a retrospective study of 945 Morse taper implants followed for one to nine years, 25 abutment-related complications occurred (2.65%), including 13 abutment fractures (1.38%) and 12 screw loosenings (1.27%), although the authors concluded that the connection was safe [33].

Deep conical engagement can also concentrate stress at the abutment neck, and the high-friction or “cold-weld” behavior that contributes to connection stability can create a clinical trade-off. The same frictional locking that limits micromovement and screw loosening may make intentional disassembly more difficult, particularly when a deeply engaged taper becomes tightly locked. This has practical chairside implications because retrieval of a tightly friction-locked Morse taper abutment may be technically demanding and may require system-specific retrieval instruments or controlled disengagement techniques. In contrast, indexed internal hexagonal connections generally provide more direct access to the abutment screw and facilitate component removal when prosthetic revision is required [33,34]. Difficult retrieval may also increase the risk of component damage and complicate subsequent prosthetic management. In addition, repeated loading can produce fretting, plastic deformation, microgap changes, or component fracture, with magnitude depending on cone angle, component geometry, material properties, and implant-system design [33,34]. The comparison of outcomes between the two connection types is summarized in Table 3.

**Table 3:** Comparison of marginal bone loss and peri-implant clinical outcomes between internal hexagonal and internal conical connections The table summarizes findings from published clinical, biomechanical, microbiological, and systematic review evidence regarding implant–abutment connection design and peri-implant outcomes [3,4,6-8,12-15,18,21,23-33]. MBL, marginal bone loss.

Outcome	Internal hexagonal	Internal conical	Evidence summary
Marginal bone loss	Slightly greater in some studies	Lower in meta-analyses and some short-term studies	Meta-analyses favor conical; RCTs often show no significant difference
Plaque index	Low with good hygiene	Low with good hygiene	Comparable; hygiene-dependent
Bleeding on probing	May occur with inflammation	Lower in some studies	Inconsistent; hygiene-dependent
Probing depth	Clinically acceptable	Comparable	Minor differences
Mucosal recession	Possible with thin tissue	Possible reduction reported	Limited evidence
Keratinized mucosa	Anatomy-dependent	Anatomy-dependent	No connection effect
Implant stability	Screw-dependent	Friction-lock	Potential biomechanical advantage (laboratory evidence)
Peri-implant disease	Mucositis/peri-implantitis may occur	Mucositis/peri-implantitis may occur	Mainly hygiene-dependent; connection a co-factor
Mechanical complications	Greater screw-loosening susceptibility	Lower micromotion; abutment fracture can occur	Clinical differences inconsistent
Implant survival	High in the cited studies	High in the cited studies	Comparable
Implant success	Clinically predictable	Clinically predictable	Both effective

Factors Modifying Clinical Outcomes

Many variables modify outcomes independently of connection type. Implant diameter and length determine the surface available for load distribution, and placement depth sets the position of the microgap relative to the crest [35]; subcrestal placement can improve the emergence profile but may position bacterial and inflammatory stimuli deeper, and a randomized trial showed that vertical implant position and peri-implant soft-tissue thickness influence marginal bone-level change [35]. Bone quality and implant location alter primary stability and load transfer, and immediate loading is feasible only with adequate primary stability and controlled occlusion, under which conical implants have shown favorable short-term outcomes [36,37].

Soft-tissue phenotype is an additional modifier of marginal bone stability. Mucosal thickness and the width of keratinized mucosa may influence peri-implant tissue remodeling and marginal bone levels, although their effects are not independent of implant position, abutment dimensions, and other patient- and implant-related factors [21,38-40]. Importantly, these soft-tissue characteristics may interact with implant-abutment connection design and platform configuration; therefore, differences in marginal bone preservation should not be attributed to connection geometry alone. Adequate peri-implant soft-tissue dimensions and appropriate maintenance may contribute to more stable peri-implant tissues irrespective of whether an internal hexagonal or internal conical connection is used.

Prosthesis design, including cantilevers, non-passive frameworks, high crown-to-implant ratios, angled abutments, and overcontoured restorations, affects both mechanical stress and access for hygiene [9,13,14,21]. A thin soft-tissue phenotype predisposes to recession and early remodeling [31], effects partly mitigated by platform switching and by an adequate transmucosal abutment height, both of which help establish a stable supracrestal attachment [11,19,31]. Oral hygiene is critical because plaque-induced inflammation can overcome any theoretical sealing advantage of a conical interface [21]. Smoking impairs vascularity and healing and increases marginal bone change [38], with a relationship between smoking frequency and more severe loss [39], and diabetes has been associated with greater marginal bone loss [40]. Follow-up duration also shapes conclusions: short studies capture healing-related remodeling, whereas longer observation reveals progressive disease, fatigue, and prosthetic complications [33], which partly explains why some data favor conical connections [24] while matched randomized trials report comparable outcomes [26].

Comparative Interpretation

Overall, the evidence suggests a distinction between mechanistic advantages and demonstrated clinical benefit. Although laboratory, finite element, and microbiological studies support potential advantages of conical connections, well-controlled randomized trials generally have not demonstrated significant differences in marginal bone loss, peri-implant health, complications, or survival between internal hexagonal and internal conical connections [25-28]. A one-year comparison also found no significant difference between screwless Morse taper and passive-fit screw-retained connections [29].

An important confounder is platform switching. Many contemporary conical implant systems incorporate a reduced-diameter abutment or platform-switched configuration, making it difficult to attribute observed marginal bone preservation to the taper alone. Platform switching itself has been associated with reduced marginal bone loss, and systematic evidence suggests that its effect may occur across different implant-abutment connection designs [3,11]. Thus, when conical implants demonstrate lower marginal bone loss, the observed benefit may reflect the combined effects of conical engagement, platform switching, implant-abutment interface position, and related system-specific design features rather than the taper geometry in isolation. Direct comparisons that hold platform configuration constant are therefore needed to determine the independent contribution of the conical connection.

The apparent differences between studies may reflect heterogeneity in implant system, taper angle, platform configuration, abutment height, radiographic methods, and follow-up duration, with some retrospective studies also comparing different implant systems [24]. In addition, small differences in marginal bone loss may have limited clinical relevance in the absence of inflammation or other peri-implant complications, although they may be more important in patients with thin tissues, limited crestal bone, high esthetic demands, or increased disease risk [39,40].

Clinical Decision-Making

Connection selection should be individualized rather than based on geometry alone, taking into account patient risk, site anatomy, tissue phenotype, loading conditions, esthetic demands, prosthetic requirements, maintenance capacity, and evidence for the specific implant system [8,21,25-28]. An internal conical connection may be considered in esthetically demanding or high-load situations when its design characteristics and system-specific evidence support its use [4,12,31,35,37]. However, potential benefits should be interpreted alongside platform configuration and other factors that may influence marginal bone preservation. For deep Morse taper systems, the benefits of frictional stability should be balanced against potential retrieval difficulty and mechanical complications when future prosthetic intervention or component replacement is anticipated [33,34].

Internal hexagonal connections remain appropriate when indexed positioning, prosthetic flexibility, component retrieval, or multi-unit alignment are important. In all cases, adequate primary stability, three-dimensional positioning, passive fit, controlled occlusion, and maintenance are more important than connection geometry alone [36]. High-risk patients, including smokers and patients with diabetes, previous periodontitis, bruxism, or inconsistent maintenance, require appropriate risk-factor control and supportive peri-implant care regardless of connection type (Figure 3) [8,21,38-40].

**Figure 3:**
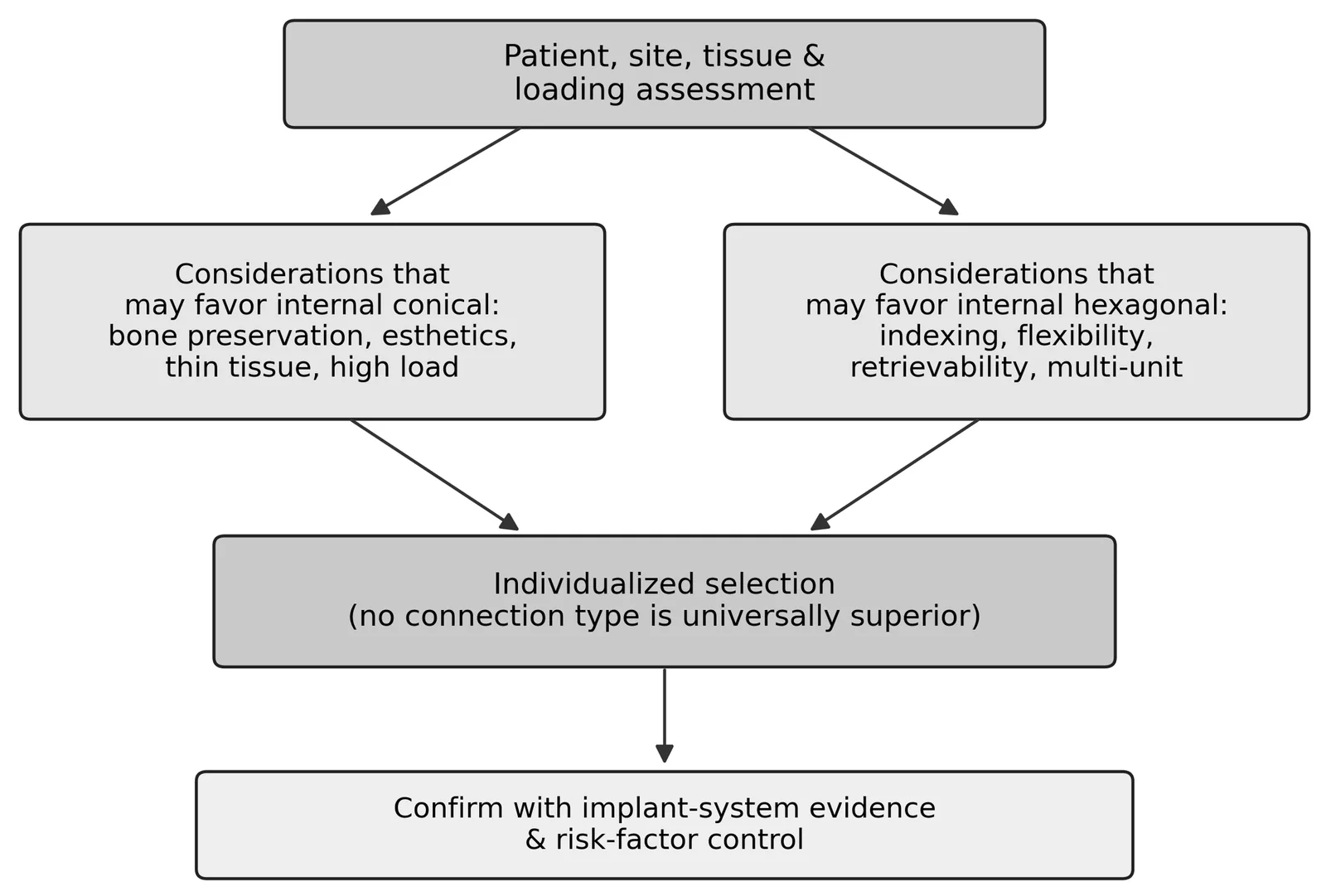
Individualized selection of implant–abutment connection based on patient, site, tissue, and loading considerations Conceptual framework for individualized selection of internal hexagonal or internal conical implant–abutment connections. Patient-, site-, tissue-, and loading-related factors may influence connection selection; no connection type is universally superior. Selection should be confirmed against evidence for the specific implant system and consideration of prosthetic requirements and modifiable risk factors.

Limitations

Several limitations apply. This is a narrative review without a formal protocol, risk-of-bias assessment, or quantitative meta-analysis, and it was restricted to English-language publications, so selection and publication bias cannot be excluded. The primary literature is heterogeneous in implant system, connection geometry, taper angle, platform configuration, abutment height, baseline definitions, radiographic method, loading protocol, and follow-up duration, and some comparisons involve different implant systems, which prevents isolation of the connection effect. Meta-analyses in this field are themselves constrained by the heterogeneity and generally short- to medium-term follow-up of their included studies. The findings should therefore be interpreted as a qualitative synthesis rather than pooled evidence.

Future Directions

Progress requires standardized baselines, radiographic methods, probing criteria, and reporting intervals, together with adequately powered randomized controlled trials that hold implant macrogeometry, surface, placement depth, abutment height, prosthesis design, and loading protocol constant so that the connection effect can be isolated, ideally with follow-up beyond five years and across multiple centers. Digital and artificial intelligence-assisted radiographic measurement, standardized peri-implant charting, and patient-reported outcomes would strengthen future comparisons, and predictive models integrating systemic risk, tissue phenotype, site, loading, and prosthetic needs could help translate evidence into individualized connection selection.

## Conclusions

Internal hexagonal and internal conical implant-abutment connections both provide high survival and predictable peri-implant outcomes. Laboratory, finite element, and microbiological studies, along with several meta-analyses, indicate that conical connections may reduce micromovement, microgap opening, and bacterial leakage, distribute load more favorably, and better preserve crestal bone in the short to medium term; however, well-controlled randomized trials, including recent evidence, generally show no significant difference between connection types in marginal bone loss, peri-implant health, complications, or survival. Connection-specific clinical superiority has therefore not been consistently established, and observed differences in clinical outcomes are small and strongly influenced by hygiene, smoking, systemic health, soft-tissue phenotype, implant position, abutment height, prosthesis design, and maintenance. Importantly, the apparent bone-preserving advantage of conical connections may partly reflect concomitant platform switching and other system-specific design features, while the high-friction locking of deep Morse taper connections may reduce retrievability and introduce mechanical management considerations. An internal conical connection may be considered when marginal bone preservation and esthetic stability are priorities, and an internal hexagonal connection when prosthetic versatility, indexed positioning, and retrievability are required, but selection should be individualized and guided by patient- and site-specific factors and by the evidence for the specific implant system rather than by connection geometry alone.
